# Ciliary Ion Channels in Polycystic Kidney Disease

**DOI:** 10.3390/cells14060459

**Published:** 2025-03-19

**Authors:** Lubna A. Alshriem, Raghad Buqaileh, Qasim Alorjani, Wissam AbouAlaiwi

**Affiliations:** 1 Department of Pharmacology and Experimental Therapeutics, University of Toledo, Toledo, OH 43614, USA; lalshri@rockets.utoledo.edu (L.A.A.); raghad.buqaileh@rockets.utoledo.edu (R.B.); qasim.alorjani@rockets.utoledo.edu (Q.A.); 2 Department of Clinical Pharmacy, Faculty of Pharmacy, Jordan University of Science and Technology, P.O. Box 3030, Irbid 22110, Jordan

**Keywords:** polycystic kidney disease, primary cilia, ion channels, polycystin, Ca^+2^, TRPV4, CFTR

## Abstract

Polycystic kidney disease (PKD) is the most common hereditary disorder that disrupts renal function and frequently progresses to end-stage renal disease. Recent advances have elucidated the critical role of primary cilia and ciliary ion channels, including transient receptor potential (TRP) channels, cystic fibrosis transmembrane conductance regulator (CFTR), and polycystin channels, in the pathogenesis of PKD. While some channels primarily function as chloride conductance channels (e.g., CFTR), others primarily regulate calcium (Ca^+2^) homeostasis. These ion channels are essential for cellular signaling and maintaining the normal kidney architecture. Dysregulation of these pathways due to genetic mutations in *PKD1* and *PKD2* leads to disrupted Ca^+2^ and cAMP signaling, aberrant fluid secretion, and uncontrolled cellular proliferation, resulting in tubular cystogenesis. Understanding the molecular mechanisms underlying these dysfunctions has opened the door for innovative therapeutic strategies, including TRPV4 activators, CFTR inhibitors, and calcimimetics, to mitigate cyst growth and preserve renal function. This review summarizes the current knowledge on the roles of ciliary ion channels in PKD pathophysiology, highlights therapeutic interventions targeting these channels, and identifies future research directions for improving patient outcomes.

## 1. Introduction

Polycystic kidney disease (PKD) is one of the most prevalent hereditary conditions and is part of a group of monogenic disorders that cause renal cyst formation. In advanced stages, PKD is the leading cause of end-stage renal failure and a common indication for dialysis or renal transplantation. The dominant form, autosomal dominant polycystic kidney disease (ADPKD), primarily affects adults [1], with an estimated 600,000 individuals diagnosed in the United States, and 12.4 million cases worldwide [2]. Approximately 7–10% of ESRD patients require hemodialysis [3]. It is characterized by gradual cyst growth in ductal organs, particularly in the kidneys and the liver, which start to develop in utero and can originate from all nephron segments of the kidneys and in the liver. The disorder is driven by genetic mutations, mainly in *PKD1* and *PKD2*, which encode polycystin-1 (PC1) and polycystin-2 (PC2), respectively [4]. Notably, *PKD1* mutations account for 85–90% of the cases and are associated with significantly more severe diseases, whereas 10–15% of ADPKD cases are attributed to mutations in *PKD2*, and those patients have a later onset of symptoms and a slower rate of progression to renal failure. There has been a notable increase in the recognition of genetic heterogeneity in PKD, highlighting the involvement of genes beyond *PKD1* and *PKD2*. Whole-genome sequencing (WGS) has revealed several additional genes associated with variants that contribute to ADPKD. These include *DNAJB11* [5], *GANAB* [6], *ALG9* [7], and more recently identified genes such as *IFT140* [8] and *ALG5* [9].

Autosomal recessive polycystic kidney disease (ARPKD) is much rarer than ADPKD; it has been estimated to have an incidence of 1 in 20,000 [10]. Autosomal recessive polycystic kidney disease (ARPKD) is caused by mutations in the *PKHD1* gene, which encodes the fibrocystin/polyductin (FPC) protein. Fibrocystin is primarily expressed in the kidneys and bile ducts, playing a crucial role in maintaining tubular and ductal structures, with its dysfunction leading to cyst formation and fibrosis [11,12]. Severely affected fetuses display a “Potter-like” oligohydramnios phenotype with lethal pulmonary hypoplasia [13]. Most newborns that survive the perinatal phase suffer renal failure and hepatic fibrosis, while a considerable percentage (30%) of cases die by the first few weeks of life. However, ARPKD can be diagnosed in childhood, adolescence, or adulthood, with less severe kidney disease and more complications of liver disease [14].

Ciliopathies are a growing group of genetic disorders affecting multiple organ systems, resulting from various mutations in genes encoding proteins essential for ciliary structure and function. Both ADPKD and ARPKD are classified as ciliopathies, as they are associated with primary cilia dysfunction.

The primary cilium is a hair-like microtubule-based organelle that extends from the surface of nearly all mammalian cells and is attached to the mother centriole [15]. These cilia have a mechanosensory function, are stimulated by the surrounding microenvironment, and translate these stimulations into intracellular signaling to produce specific responses [16]. The primary cilia have a unique structure that consists in a basal body acting as a genesis site for the microtubules, which form the functional structure of the cilia, the axoneme. The primary cilia length extends from 2 to 10 μm, reaching 200 μm in olfactory neurons [17]. The ciliary membrane is extended from the plasma membrane and houses several unique receptors, proteins, ion channels, and signaling molecules that can be activated by mechanical or chemical stimuli to regulate various cellular functions, and their intracellular signaling is tissue-specific [18,19]. Ciliary ion channels are believed to be central to maintaining intracellular homeostasis and the structural integrity of the kidney tissue. Ion channels like transient receptor potential (TRP) channels, including TRPM4, TRPV4, TRPC1, and PC2, function as Ca^+2^-permeable ion channels, and their impaired regulation may lead to altered intracellular Ca^+2^ signaling. In PKD, renal epithelial cells are characterized by decreased intracellular Ca^+2^ [20], which contributes to cyst formation and expansion [21] and is proposed to be the primary causative abnormality in PKD pathogenesis [22]. Thus, understanding the specific functions and mechanisms of action of ciliary ion channels can provide significant insights into the pathophysiology of PKD. This knowledge can potentially guide the development of targeted therapies aimed at correcting defective ion channel function or modulating the downstream signaling pathways that they regulate. Such advances could lead to novel treatments that slow the progression of cyst development, ultimately preserving kidney function and improving patient outcomes.

## 2. Role of Ca^+2^ in the Pathogenesis of PKD

PKD is associated with dysregulated Ca^+2^ signaling, which plays a critical role in disease progression. Impaired polycystin function leads to decreased intracellular Ca^+2^ levels, disrupting Ca^+2^-dependent signaling pathways. This shift results in excessive cyclic adenosine monophosphate (cAMP) production, which promotes cyst formation and fluid secretion. Primary cilia have been reported to regulate the influx of Ca^+2^ ions through the TRP channels, including polycystins, which function as fluid flow mechanosensors [23,24]. However, it has been shown that the primary cilia are not Ca^+2^ -responsive mechanosensors, as cilia-specific Ca^+2^ influxes were not observed in the presence of physiological and highly supraphysiological levels of fluid flow. This study demonstrated that the initial Ca^+2^ oscillation occurs in the cytoplasm in response to flow, which then diffuses into the cilia [25]. Therefore, the role of ciliary mechanosensation in managing Ca^+2^ regulation remains a topic of ongoing debate.

**Figure 1:**
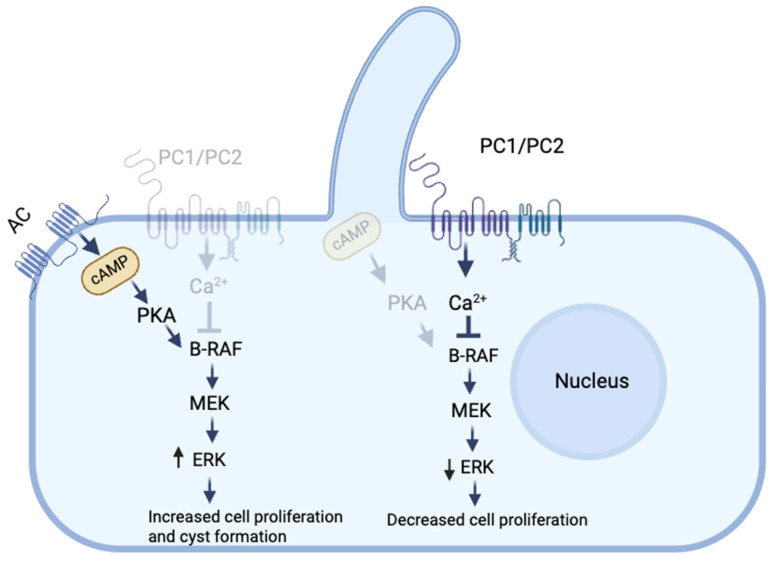
Cyclic adenosine monophosphate (cAMP) signaling with functional and dysfunctional polycystin. Functional polycystin maintains normal intracellular Ca^+2^ levels, which inhibit B-rapidly accelerated fibrosarcoma (B-RAF) activity, leading to reduced mitogen-activated protein kinase (MEK) and extracellular signal-regulated kinase (ERK) signaling that suppresses cell proliferation and supports normal tubule formation. In PKD, dysfunctional polycystin leads to cAMP-mediated activation of protein kinase A (PKA)/B-RAF/MEK/ERK, promoting increased cell proliferation and cyst formation. Transparent receptors and pathways: diminished pathways. Ca^+2^: calcium, AC: adenylyl cyclase. Created by BioRender.com.

Depending on the cell type, cAMP can either be mitogenic or anti-mitogenic. cAMP plays a key role in cyst formation by promoting fluid secretion and cell proliferation [26]. It was found that cAMP inhibited the growth of normal renal epithelial cells and stimulated the growth of cells derived from the cysts of PKD patients. These studies showed that the treatment of cultured cells derived from the cysts of PKD patients with cAMP increased the rate of cell proliferation through the sequential phosphorylation of protein kinase A (PKA), B-rapidly accelerated fibrosarcoma (B-Raf), mitogen-activated protein kinase (MEK), and extracellular signal-regulated kinase (ERK) [27] (Figure 1). It is believed that PC1 and PC2 are part of a multiprotein signaling complex that controls the intracellular Ca^+2^ levels in response to external signals [28]. This suggests that impaired polycystin function may disrupt Ca^+2^ signaling, leading to a shift toward a cAMP-dependent growth-stimulated phenotype. Yamaguchi et al. studied the effect of Ca^+2^ channel blockers on mouse M-1 collecting duct cells and primary cultures of human kidney epithelial cells, and the results showed that Ca^+2^ channel blocking converted both cell types from a normal cAMP-growth-inhibited phenotype to an abnormal cAMP-growth-stimulated phenotype, characteristic of PKD [29]. Furthermore, maintaining a sustained increase in intracellular Ca^+2^ levels completely reversed the mitogenic response to cAMP. Moreover, it has been shown that treatment of ADPKD or ARPKD cells with either a Ca^+2^ channel activator (Bay K8644) or a Ca^+2^ ionophore (A23187) increased the intracellular Ca^+2^ levels and restored the normal anti-mitogenic response to cAMP [20].

In the Ca^+2^-deficient environment of PKD mutant cells, it has been proposed that Ca^+2^-regulated adenylate cyclases (ACs), which are enzymes that convert ATP to cAMP, and Ca^2+^-regulated phosphodiesterases (PDEs), which hydrolyze cAMP, may play a role in elevating cAMP levels. Studies have shown that ADPKD cells have high expression of the Ca^+2^-inhibited AC5 and 6, suggesting that the high AC levels induce the production of cAMP and induce cyst growth in response to low Ca^+2^ levels [30]. This conclusion was supported by studies investigating the role of AC6 in a collecting-duct-specific *Pkd1* knockout mouse model and a *Pkd1*/AC6 double-knockout mouse model of PKD. *Pkd1* knockout led to severe kidney failure, massive cyst formation, and death within two months. However, the simultaneous knockout of *Pkd1* and AC6 significantly reduced cystogenesis, improved kidney function, suppressed the B-Raf/ERK/MEK pathway, and extended survival. Furthermore, the findings indicated that the targeted knockout of AC6 did not impact the overall kidney or urinary cAMP levels, implying that decreasing cAMP, specifically within cyst cells, was adequate to inhibit cyst growth [31].

## 3. Ciliary Ion Channels

Ciliary ion channels play crucial sensory and signaling roles and are involved in detecting mechanical and chemical stimuli in the environment, contributing to the regulation of ion flow and cellular homeostasis [32]. Some of the most significant types of ion channels found in primary cilia include TRP channels, cystic fibrosis transmembrane conductance regulator (CFTR), and polycystin channels [33]. They are localized in the cell membrane and endoplasmic reticulum (ER) [34]. The TRP family ion channels function as molecular sensors by regulating Ca^+2^ influx. Ca^+2^ signaling and intracellular homeostasis are essential factors that determine cellular survival and function. Cystogenesis has been directly associated with the disruption of intracellular Ca^+2^ homeostasis. It was reported that intracellular Ca^+2^ homeostasis is disrupted in ADPKD and ARPKD, as demonstrated by a reduction in intracellular Ca^+2^ levels, Ca^+2^ stores, and Ca^+2^ channel activities [29]. In addition to the primary involvement of Ca^+2^ in PKD, studies addressed the role of chloride ions in fluid secretion and cyst formation [35]. The primary mechanism underlying cAMP-induced transmembrane fluid secretion in ADPKD has been found to be CFTR-mediated chloride transport [36]. Studies conducted both in vitro and in vivo demonstrated that CFTR is necessary for the development and growth of cAMP-stimulated cysts [37,38,39]. Therefore, it is believed that cyst formation and fluid accumulation in renal cysts are caused by the disruption of intracellular Ca^+2^ homeostasis through dysfunctional TRP channels and active chloride secretion through CFTR, which is stimulated by the cAMP/PKA pathway [40].

### 3.1. TRP Channels

The TRP channels are a family of diverse ion channels that mediate the transmembrane flux of cations down their electrochemical gradients, thereby raising the intracellular Ca^+2^ and Na^+^ concentrations and depolarizing the cell membrane. The TRP superfamily consists of six subfamilies, including canonical (TRPC), vanilloid (TRPV), melastin (TRPM), ankyrin (TRPA), mucolipin (TRPML), and polycystin (TRPP) channels [41].

#### 3.1.1. Polycystins

PC1 is a large transmembrane protein belonging to the transient receptor potential (TRP) family and is composed of an extracellular (N-terminal) domain, a multiple transmembrane domain, and a cytoplasmic (C-terminal) domain. The N-terminal domain contains several structural components essential for interactions with extracellular proteins [42]. Additionally, the C-terminus features a binding site for heterotrimeric G proteins, facilitating the activation and regulation of G protein-mediated signaling pathways [43,44,45].

**Figure 2:**
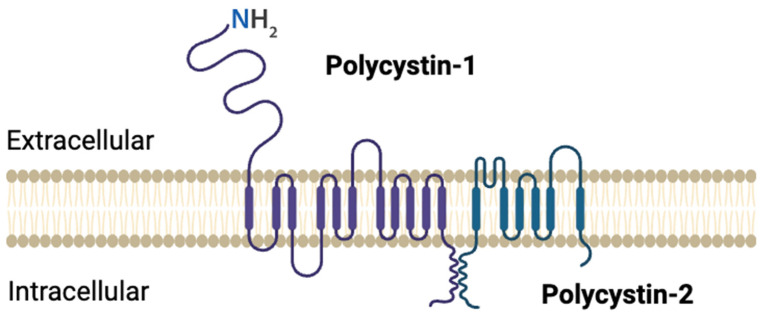
Structural overview of polycystin 1 (PC1) and polycystin 2 (PC2). Created by BioRender.com.

PC2 structure consists of six transmembrane domains with a cytosolic C- and an extracellular N-terminus [46]; it is a non-selective Ca^+2^-permeable channel that helps in maintaining intracellular Ca^+2^ homeostasis and regulating intracellular signaling cascades. Figure 2. Shows the structure of PC1 and PC2. PC2 has been suggested/demonstrated to form a complex with PC1 [47] and functions independently or as a component of the PC1/PC2 complex [48]. It has been shown that PC1 is critical for the functioning of the PC1/PC2 complex [49]. However, one study showed that PC1 can negatively affect ion transport through the complex. This is related to the presence of three positively charged residues—Arg4100, Arg4107, and His4111—that extend into the cavity of the ion-conducting pathway, potentially obstructing the permeability of the Ca^+2^ ion. Therefore, the structure could represent a potentially non-conductive state [50].

Both PC1 and PC2 interact with inositol 1,4,5-trisphosphate receptor (IP_3_R) in the endoplasmic reticulum (ER) to regulate intracellular Ca^+2^. While PC2 enhances Ca^+2^ release from the ER by stimulating the activity of IP_3_R, PC1 inhibits this process by reducing the PC2-IP_3_R interaction via a mechanism involving stromal interaction molecule-1 (STIM1) and the PI3K/Akt pathway. It has been shown that in cells harboring the *Pkd1* mutation, inhibition of the PI3K/Akt pathway strengthened the connection between PC2 and IP_3_R, disrupted the STIM1-IP_3_R complex, and restored intracellular Ca^+2^ release [51]. The external C-terminal fragment of PC1 may act as a dominant negative effector, resulting in enhanced intracellular calcium release in response to ATP treatment, as observed in HEK-293 cells [52]. Moreover, cells expressing PC1 C-terminus not only displayed elevated levels of basal calcium but also demonstrated increased cell proliferation. This enhanced proliferation was linked to the activation of ERK kinases [53]. Transfecting HEK-293 cells with the C-terminal tail of PC1 has been shown to elevate both basal and intracellular calcium release, resulting in the activation of nuclear factor of activated T-cells (NFAT) [54].

PC1 interacts and regulates other types of Ca^+2^ channels, including non-capacitative Ca^+2^ entry (NCCE) channels, which can generate intracellular Ca^+2^ oscillations with downstream effects on cell proliferation. Aguiari et al. investigated the role of PC1 in modulating NCCE and Ca^+2^ oscillations, with downstream effects on cell proliferation. The results showed that the depletion of endogenous PC1 in HEK293 cells increased serum-induced Ca^+2^ oscillations and cell proliferation, while PC1-mutated kidney cystic cell lines exhibited similar abnormalities, which were reduced by exogenous PC1 expression, highlighting PC1 role in regulating Ca^+2^ homeostasis and cell cycle progression in ADPKD [55].

**Figure 3:**
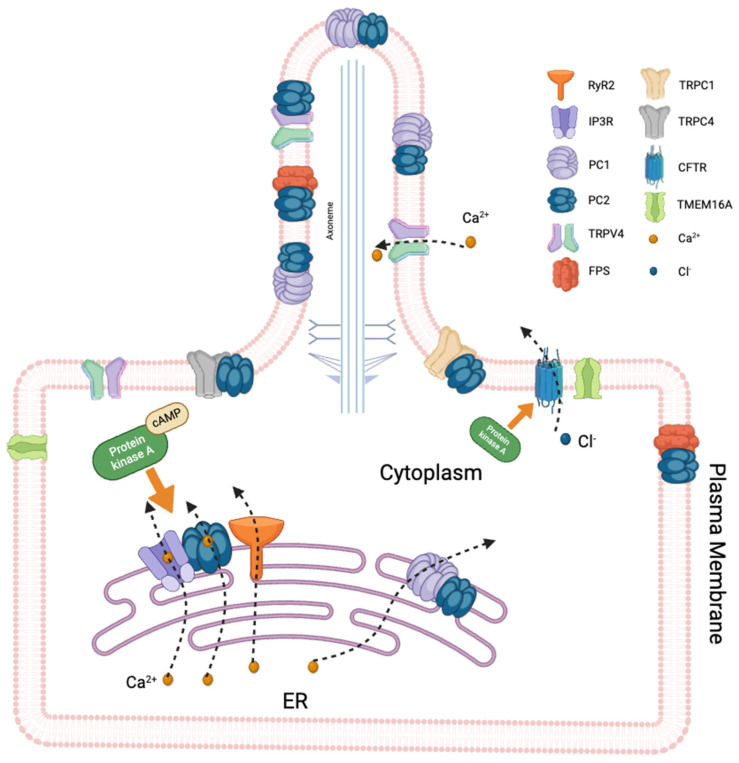
Schematic representation of ciliary and cell membrane ion channels involved in signal transduction. The illustration highlights polycystin-1 (PC1) and polycystin-2 (PC2) within the primary cilium, mediating Ca^+2^ signaling alongside other ion channels in the cilium and the cell membrane, regulating the ion flux and cellular homeostasis. Dysfunction in these channels is linked to cystogenesis in polycystic kidney disease (PKD). Elevated intracellular cAMP levels activate PKA, leading to the activation and increased gating activity of inositol 1,4,5-trisphosphate receptor (IP3R), ryanodine receptor (RyR), and PC2 within the endoplasmic reticulum (ER). This results in a constitutive Ca^+2^ leak and subsequent depletion of intracellular Ca^+2^ stores, contributing to PKD pathophysiology. CFTR: cystic fibrosis transmembrane conductance regulator, TRPC1: transient receptor potential canonical 1, TRPC4: transient receptor potential canonical 4, FPS: fibrocystin, TMEM16A: transmembrane member 16A, anoctamin 1, Ca^+2^: calcium, Cl^−^: chloride. Created by BioRender.com.

The function of the PC2 ion channel was studied independently of that of PC1 in transgenic mice, and it was demonstrated that the PC2 ion channel forms a functional ion channel in primary cilia independent of PC1 [56]. Beyond its role as a channel, PC2 interacts with and regulates several other Ca^+2^ channels, thereby influencing intracellular signaling pathways and modulating intracellular Ca^+2^ homeostasis. PC2 interacts with major intracellular Ca^+2^ release channels in the ER, such as IP3R and ryanodine receptor (RyR), thereby modulating Ca^+2^ release. Biochemical assays showed that the N-terminus of PC2 interacts with RyR2, while the C-terminus binds to RyR2 exclusively when it is in its open state. The C-terminus of PC2 functionally inhibited RyR2 channel activity in the presence of Ca^+2^. *PKD2*-knockout cardiomyocytes exhibited a higher frequency of spontaneous Ca^+2^ oscillations, reduced Ca^+2^ release from the sarcoplasmic reticulum stores, and reduced Ca^+2^ content compared with wildtype cardiomyocytes. These findings suggest that PC2 is important for the regulation of RyR2 function and that loss of RyR2 regulation results in altered Ca^+2^ signaling in the heart [57]. Another mechanism has been proposed for the depletion of the Ca^+2^ stores in PKD. High intracellular cAMP levels in PKD activate PKA, which phosphorylates and increases the gating activity of IP3R, RyR2, and PC2 in the ER. Constitutive activation of PC2, RyR2, and IP3R results in a Ca^+2^ leak and in the depletion of the intracellular Ca^+2^ stores in PKD [58] (Figure 3). Furthermore, PC2 physically and functionally interacts with IP3R through its C-terminus and modulates intracellular Ca^+2^ signaling. Studies showed that mutations in *Pkd2* could result in the dysregulation of intracellular Ca^+2^ signaling, which in turn could contribute to the pathology of PKD [59,60]. Various studies suggest that PC2 expression and function could be modulated by aging [61,62]. A recent study showed that aging disrupts the functional interaction between PC2 and RyRs, which might contribute to accelerated disease progression in elderly ADPKD patients [60].

Ciliary PC2 has been shown to play crucial roles in mediating fluid shear sensing, as well as the transduction of these mechanical signals into changes in Ca^+2^ signaling and nitric oxide (NO) synthesis in endothelial cells. A study used *Pkd2*-deficient mouse endothelial cells (*Pkd2^+/−^* and *Pkd2^−/−^*) and compared their fluid-sensing capabilities. The results showed that *Pkd2^−^/^−^* endothelial cells did not respond to fluid shear stress, while *Pkd2^+/−^* cells showed cytosolic Ca^+2^ increases in response to fluid shear. This study also confirmed that PC2 is a shear-sensitive Ca^+2^ channel that is required to activate a biochemical cascade for NO production [63]. PKD patients suffer from extra-renal manifestations including hypertension. It was proposed that the low nitric oxide production in endothelial cells from PKD patients could be associated with hypertension manifestations, demonstrating the role of vascular endothelial PC2 in blood pressure regulation [63]. PC2 has also been shown to form complexes with other TRP family members, such as TRPC1, TRPC4, and TRPV4, functioning as molecular sensors for regulating Ca^+2^ influx. In vitro assays showed that PC2 can directly associate with TRPC1 but not TRPC3 in hemagglutinin epitope-tagged PKD2 (HA-PKD2)-transfected cells. These results provide evidence that PC2 may be functionally related to TRPC proteins and provide a possible role for PC2 in regulating Ca^+2^ entry in response to Ca^+2^ store depletion and/or G protein-coupled receptor activation [64]. A study was conducted to investigate the binding of PC2 to other Ca^+2^ channels and contribute to angiotensin II (ANG II)-induced Ca^+2^ signaling in mesangial cells (MCs). The results showed that ANG II stimulation increased the expression of PC2 on the cell surface of MCs and its interaction with TRPC1 and TRPC4. Additionally, PKD2-knockdown MCs showed a substantial reduction in ANG II-induced MC contraction. These findings imply that PC2 forms channel complexes with TRPC1 and TRPC4 in a selective manner, modulating the Ca^+2^ responses in MCs generated by ANG II [65]. Recent studies showed an interaction of PC2 with IP3R in the ER [66,67]. Deng et al. showed that when the IP3 receptor is activated, it causes a reduction in the ER Ca^+2^ levels, which is detected by STIM1. In response, STIM1 activates Ca^+2^ release-activated Ca^+2^ channel modulator protein 1 (Orai1) in the plasma membrane, along with other potential Ca^+2^ channels. Ca^+2^ entering the cytosol is then transported back into the ER via the sarco/ER Ca^+2^-ATPase (SERCA) pump [68]. Recent research has shown that elevated levels of STIM1 and IP3R contribute to cyst growth by increasing the cAMP levels and promoting Ca^+2^ release from the ER, as demonstrated using STIM1 knockdown and *pkd1* knockout in an ADPKD mouse model compared to non-cystic controls [69]. Additionally, PC2 has been found to interact with TRPV4, forming a mechanosensitive and thermosensitive molecular sensor within the cilium. TRPV4 is a crucial part of the ciliary mechanosensory system, as evidenced by the elimination of flow-induced Ca^+2^ transients in renal epithelial cells upon TRPV4 depletion [70].

#### 3.1.2. TRPV4

The TRPV4 ion channel is a non-selective cation channel (Ca^+2^, Mg^+2^, or Na^+^) characterized by a moderately high Ca^+2^ permeability ratio [71]. TRPV4 can be activated by a range of chemical, osmotic, and mechanical stimuli. TRPV4 activity was significantly impaired in isolated murine collecting duct (CD)-derived cyst monolayers, with abnormal subcellular localization of the channel leading to decreased basal Ca^+2^ levels and loss of flow-mediated Ca^+2^ signaling. The long-term systemic administration of a specific TRPV4 activator in ARPKD PCK453 rats resulted in a time-dependent reduction in renal symptoms associated with ARPKD. This selective activation of TRPV4 restored its function and subcellular distribution, as well as mechanosensitive Ca^+2^ signaling at the cellular level [21]. TRPV4 function and its role in Ca^+2^ regulation were evaluated in ADPKD human kidneys and normal regions of human kidneys collected from nephrectomy specimens that were removed to treat renal carcinomas or withheld from transplantation, which were referred to as normal human kidneys (NHKs). In ADPKD cells, TRPV4 activity and TRPV4-dependent Ca^+2^ influxes were significantly decreased, which was associated with altered Ca^+2^ signaling. The results also showed a significant reduction in TRPV4 glycosylation in ADPKD cells, although the total TRPV4 protein levels were similar in NHK and ADPKD cells [72]. TRPV4 was found to interact and co-localize with PC2 on the primary cilia of polarized Madin–Darby canine kidney (MDCK) cells [70]. The expression of TRPV4 significantly enhanced the surface expression of TRPP2 (PC2), while TRPP2 (PC2) decreased both the surface expression and the total protein levels of TRPV4. Altered channel properties caused this reduction in TRPV4 expression by forming TRPP2/TRPV4 heteromers rather than by inducing an actual reduction in TRPV4 channels. These findings address the fact that TRPP2 (PC2) and TRPV4 physically and functionally interact [70]. While TRPV4 is a crucial element of ciliary mechanosensitivity in MDCK cells in vitro, it does not have a significant role in cyst formation in TRPV4-deficient mice or zebrafish [70,73]. However, Taniguchi et al. found that flow-dependent potassium secretion is a Ca^+2^-dependent process that is eliminated in TRPV4 knockout mice. Their findings imply that flow-induced Ca^+2^ transients are absent in the distal nephrons of adult TRPV4-deficient mice, reinforcing the sensory function of TRPV4 [74].

### 3.2. Cystic Fibrosis Transmembrane Conductance Regulator (CFTR)

Cystic fibrosis transmembrane conductance regulator (CFTR) is a chloride and bicarbonate ion channel encoded by the CFTR gene on chromosome 7. It is primarily expressed in epithelial cells in various tissues, including the lungs, pancreas, intestines, sweat glands, and kidneys [75]. Structurally, CFTR consists of two transmembrane domains, two nucleotide-binding domains (NBDs), and a regulatory (R) domain. The channel’s activity is regulated by phosphorylation of the R domain via protein kinase A (PKA) and the hydrolysis of ATP at the NBDs [76]. CFTR is part of the ATP-binding cassette (ABC) transporter family and operates as an anion channel essential for regulating salt and water movement across epithelial cells [75]. CFTR is expressed on the apical membrane of renal epithelial cells and may interact with components of the primary cilia. The cilium is a highly compartmentalized structure, and CFTR-mediated chloride and bicarbonate ion transport ensures proper electrochemical gradients required for ciliary signaling and mechanotransduction [77]. Interestingly, studies have shown that the absence of CFTR in the kidney epithelium leads to the elongation of primary cilia. This suggests that CFTR may play a regulatory role in controlling ciliary length. Abnormally elongated cilia are associated with disrupted signaling and mechanosensation, which can contribute to cyst formation in the kidneys. These findings highlight CFTR involvement in maintaining both the structure and the function of primary cilia [77,78]. In PKD, mutations in *Pkd1* or *Pkd2* impair ciliary function and disrupt the regulatory interactions between these proteins and CFTR. Dysfunctional CFTR exacerbates chloride ion dysregulation, leading to abnormal fluid accumulation in the renal tubules. The resulting osmotic imbalance promotes cyst expansion by drawing water into the cyst lumen [79]. Additionally, the elongation of primary cilia in the absence of CFTR further impairs signaling pathways, such as those mediated by calcium ions, which are crucial for cell cycle regulation and fluid balance. This combination of ion imbalance and disrupted ciliary signaling accelerates cystogenesis and contributes to disease progression [78]. Other studies indicate that CFTR may not be directly localized on cilia and demonstrate that although CFTR is not directly localized on cilia, it still plays a major role in regulating ciliary function by modulating ion and fluid flow in the ciliary environment [79,80]. In PKD, elevated cAMP activates PKA, which in turn activates the CFTR channel to facilitate the movement of chloride ions (Cl^−^) into the cyst lumen. This chloride ion secretion drives osmotic water transport into the cysts, causing fluid accumulation and cyst expansion [81,82]. It has been shown that CFTR inhibitors such as those belonging to the thiazolidinone and glycine hydrazide classes slowed cyst expansion in in vitro and in vivo models of PKD. In an embryonic kidney cyst model, these compounds reduced both the number and the growth of cysts by over 80%. Furthermore, the treatment of mice for up to 7 days with these compounds significantly delayed kidney enlargement and cyst growth, while maintaining the renal function [83]. The epithelial sodium channel (ENaC) was found to co-localize with CFTR in the apical membrane and is responsible for sodium reabsorption. Its dysregulation in PKD is linked to abnormal fluid secretion and cyst enlargement [84]. cAMP can upregulate ENaC activity, leading to increased sodium reabsorption. However, in cystic epithelial cells, the dysregulation of ENaC, combined with altered Cl^−^ transport (e.g., through CFTR), contributes to excessive fluid accumulation in cysts. In *Pkd1*-knockout mice, ENaC showed reduced co-localization with plasma membrane CFTR in cysts, which aligns with their inability to absorb fluid. The treatment with VX-809 (a treatment that increased the CFTR levels at the apical membrane and reduced its association with the endoplasmic reticulum) of cystic mice elevated ENaC levels at the apical plasma membrane, supporting fluid absorption [82].

Moreover, transmembrane member 16A anoctamin 1 (TMEM16A) is a Ca^+2^-activated Cl^−^ channel that is essential for fluid secretion into renal cysts in vitro [85,86]. Studies showed that loss of *pkd1* led to increased expression of TMEM16A and CFTR, as well as enhanced Cl^−^ secretion in murine kidneys, with TMEM16A playing a key role in promoting cyst growth. Upregulated TMEM16A enhanced intracellular Ca^+2^ signaling and induced cyst growth, while TMEM16A knockdown resulted in the normalization of Ca^+2^ signaling and cell proliferation. These findings established upregulated TMEM16A as a crucial factor in the progression of ADPKD [87].

### 3.3. Fibrocystin

Fibrocystin (FPC) is a protein encoded by the polycystic kidney and hepatic disease 1 (PKHD1) gene, whose mutation results in ARPKD. FPC is co-localized with PC2 in the primary cilium/basal body and plasma membrane in renal epithelium cells [88,89,90]. A lack of FPC was found to be associated with the downregulation of PC2 expression in vivo. Renal cyst formation was significantly more pronounced in mice with mutations in both *pkhd1* and *pkd2* compared to those with only a heterozygous *pkd2* mutation. Furthermore, PC2 channel activity was disrupted in primary renal epithelial cell cultures from *Pkhd1* mutant mice. These observations indicate that FPC and PC2 interact functionally and operate within the same molecular pathway [91]. Based on these observations and the established interaction between FPC and PC2, it was proposed that FPC has a role in regulating the intracellular Ca^+2^ levels, perhaps by controlling PC2 activity. Wang et al. demonstrated that blocking the extracellular epitopes of FPC blocked cellular Ca^+2^ responses to flow stimulation [92]. Several studies indicate that FPC downregulation leads to a decrease in intracellular Ca^+2^ levels. Fibrocystin likely maintains intracellular Ca^+2^ homeostasis by modulating PC2 channel activity and preventing its degradation [91,93,94].

## 4. Therapeutic Implications: Targeting Ciliary Ion Channels in PKD

Ciliary ion channels, particularly PC2, are crucial for regulating Ca^+2^ influx into cells. The disruption of this process in PKD leads to abnormal cellular behaviors, including increased proliferation, altered fluid secretion, and excessive activation of secondary signaling pathways such as the cAMP-mediated pathway. The hyperactivation of cAMP-mediated pathways contributes to cyst growth by promoting fluid secretion and cell proliferation [26]. Modulating these pathways has emerged as a key approach for therapeutic interventions. One approach involves directly targeting PC2 to restore its Ca^+2^ channel activity. Small molecules that stabilize or enhance the function of PC2 are under investigation. Triptolide, a natural product isolated from the traditional Chinese medicine Lei Gong Teng, induced Ca^+2^ release through a PC2-dependent pathway in kidney epithelial cell lines derived from *Pkd^+/−^* or *Pkd^−/−^* mice. Furthermore, in a murine model of ADPKD, triptolide inhibited cell proliferation and reduced cyst formation by restoring Ca^+2^ signaling in kidney epithelial cells [95]. Moreover, calcimimetics, which are allosteric modulators of the Ca^+2^-sensing receptor, were investigated in vivo to evaluate their efficacy in managing PKD. The findings indicated that calcimimetics decreased cyst growth by enhancing the intracellular Ca^+2^ levels in late-stage PKD [96].

In vitro and in vivo studies suggest that increasing intracellular Ca^+2^ by TRPV4 activation may represent a potential therapeutic approach in PKD. Activation of TRPV4 by 4αPDD and GSK1016790 raises the intracellular Ca^+2^ levels, which leads to the inhibition of cholangiocyte proliferation in vitro and a reduction in cyst growth in vivo, mediated by the Akt and B-RAF/ERK1/2 signaling pathways [97]. Additionally, other ion channels, such as TMEM16A and CFTR, were identified as key players in fluid secretion into renal cysts during in vitro studies. These studies strongly suggest TMEM16A as a therapeutic target in ADPKD. A study showed that the TMEM16A inhibitors niclosamide and benzbromarone suppressed cyst growth in *Pkd1^−/−^*/T16a^−/−^ mice in vivo [87]. This study also showed that Ani9, which is a small molecule that specifically interferes with and inhibits the TMEM16A channels, led to the inhibition of cystogenesis in PKD model mice [87,98]. VX-809, a corrector of CFTR, was shown to slow cyst growth both in vivo and in vitro and improve the renal function in *Pkd1*^−/−^ mice. VX-809 showed multiple mechanisms of action, including down-regulating cAMP levels, which reduced cell proliferation. It also affected AC3 activity, leading to a decrease in resting intracellular Ca^+2^ levels and the release of Ca^+2^ from the endoplasmic reticulum [99]. Furthermore, steviol has been found to reduce MDCK cyst formation and growth by directly inhibiting CFTR chloride channel activity and reducing CFTR expression, particularly by promoting the proteasomal degradation of CFTR [100]. Another strategy involves the indirect modulation of ciliary ion channel activity by targeting the cAMP pathway. Vasopressin receptor antagonists, such as tolvaptan, have been shown to reduce the cAMP levels, thereby mitigating cyst growth [101]. While not specific to ciliary ion channels, these therapies highlight the interconnected nature of signaling pathways in PKD. The heterogeneity of PKD and the complexity of ciliary signaling pathways necessitate a multifaceted approach to therapy. Future research should focus on understanding the precise mechanisms of ciliary ion channel dysfunction and identifying safe and effective modulators. With continued exploration, targeting ciliary ion channels may offer a pathway to innovative treatments for PKD and improved outcomes for patients. Table 1 summarizes the therapeutic interventions that target ciliary ion channels in PKD.

## 5. Challenges of Studying and Targeting Ciliary Ion Channels

In PKD, ion channel dysfunction leads to abnormal cellular responses, including unregulated cell proliferation, fluid secretion into cysts, and impaired cyst resorption. The selective targeting of ciliary ion channels poses several challenges due to the complexity and uniqueness of cilia. These challenges include the complex interplay between various ion channels and cellular signaling pathways, the genetic heterogeneity of the disease, and the localization of these ion channels in different parts of the cell. As mentioned earlier in this review, most ion channels localize to the ER when it overexpressed. An in vivo study demonstrated that PC2, expressed in the ER of epithelial cells in addition to the primary cilia membrane, functions as a Ca^+2^-activated high-conductance channel that allows for the flow of divalent cations. Elevated intracellular Ca^+2^ levels trigger the release of Ca^+2^ from internal stores via PC2 [103]. Previous studies that measured ER PC2 activity have caused confusion about the fundamental properties of the channel, such as its ion selectivity and conductance. This has led to uncertainty about whether the same channel was being tested across different experiments [104,105]. However, isolating and studying ciliary ion channels requires highly sensitive techniques that can effectively separate and analyze the ciliary membrane from the rest of the cell’s components [106].

Nanoparticles have shown promise in overcoming some of these challenges. By utilizing nanoparticles, it is possible to selectively target ciliary receptors or proteins that are involved in signal transduction and other ciliary functions. A cilia-targeted delivery system has been designed to deliver fenoldopam specifically to the primary cilia for the treatment of hypertension associated with PKD. An iron oxide nanoparticle (Fe_2_O_3_-NP)-based technology was employed, and live imaging confirmed that Fe_2_O_3_-NPs specifically targeted the primary cilia both in vitro and in vivo [107]. Furthermore, polymeric nanoparticles (PNPs) loaded with fenoldopam have been designed and investigated in a ciliopathic hypertensive mouse model. The results showed that the PNPs significantly improved cardiac function and corrected the exhibited arrhythmia. These results highlighted the significant clinical potential of nanoparticles for ciliotherapy [108]. However, the design of nanoparticles that can effectively navigate to and across the ciliary membrane while minimizing off-target effects is still a developing area of research. Additionally, the need for high specificity to avoid interactions with non-ciliary cells remains a critical issue.

## 6. Future Directions

A promising future direction in PKD research is the development of ion channel modulators to restore the normal function of ciliary ion channels. This strategy could involve the use of small molecules, biologics, or other therapeutic agents that specifically target defective ion channels, such as TRPP2 (PC2) [109]. By modulating the activity of these ion channels, it may be possible to normalize the intracellular calcium levels and restore a proper fluid balance in the renal tubules. The selective modulation of these ion channels could offer a targeted treatment approach that avoids broader systemic effects, reducing the side effects associated with the current treatments [110]. Another promising direction is gene therapy, which could provide a long-term solution for patients with PKD caused by defective ciliary ion channels. Given that mutations in the *PKD1* and *PKD2* genes result in the dysfunction of TRPP2 (PC2) and other ion channels, replacing or repairing these genes could restore the normal channel function. Advances in gene-editing technologies, such as CRISPR-Cas9, could allow researchers to directly correct mutations in the *PKD1* or *PKD2* genes in renal cells [111,112]. However, many challenges are associated with the CRISPR-Cas9 technology. One significant hurdle is the large size of the PKD1 gene, which complicates its effective packaging into delivery systems for gene editing [113].

Additionally, the presence of multiple PKD1 pseudogenes can lead to off-target effects, where CRISPR inadvertently alters unintended genomic regions, potentially leading to harmful consequences [114,115]. The efficient delivery of CRISPR components to renal cells is another challenge. The current methods, such as viral vectors and lipid nanoparticles, may face limitations regarding specificity and efficiency, as well as the potential induction of immune responses [116]. Furthermore, ensuring that gene editing occurs exclusively in renal cells is critical, as non-specific editing can result in adverse effects in other cell types [117].

Alternatively, gene therapy approaches could focus on delivering healthy copies of these genes to the affected tissues to restore the normal ciliary function. By correcting the underlying genetic defect, this approach could potentially impede the progression of PKD or perhaps reverse cyst formation, offering a treatment option for patients with genetic forms of the disease [118]. However, challenges in delivering the correct genetic material to kidney cells, ensuring its stable expression and avoiding immune responses, remain significant obstacles. Continued research on efficient delivery systems and the long-term safety of gene therapy will be crucial to the success of this strategy [119].

## 7. Conclusions

Ciliary ion channels play a crucial role in maintaining kidney function by regulating essential cellular processes in primary cilia. Disruptions in these channels have been strongly implicated in the progression of PKD, highlighting their importance in disease pathology. Understanding the precise mechanisms by which ion channel dysfunction contributes to PKD offers valuable insights into disease progression and potential therapeutic targets. Ongoing research into the physiology of ion channels in primary cilia is essential to uncover new dimensions of their function and regulation. By elucidating the intricate relationships between ciliary ion channels and PKD pathology, researchers can identify novel therapeutic strategies aimed at restoring ion channel function. These advances hold the potential to significantly improve the outcomes for patients affected by PKD, paving the way for innovative and targeted treatments.

## Figures and Tables

**Table 1:** Summary of therapeutic interventions that target ciliary ion channels in PKD.

Drug	Target and MOA	Pre-Clinical Outcome	Clinical Trial Outcome
Triptolide	Induces Ca^+2^ release through a PC2-dependent pathway [95] Cell cycle arrest in G0 phase [102]	Triptolide was shown in experimental studies to inhibit cyst formation and growth [95]	Triptolide demonstrated effectiveness in managing PKD, as evidenced by its association with a significant reduction in proteinuria in PKD patients during an uncontrolled clinical trial. However, the volume of polycystic kidney and eGFR were not influenced [102]
R-568	Calcium-sensing receptor	R-568 inhibited cyst growth and fibrosis in late stage PKD	-
4αPDD and GSK1016790	TRPV4 activator	TRPV4 activation in PKD cholangiocytes increased [Ca^+2^]i by 30%, inhibiting cell proliferation by approximately 25–50% and cyst growth in 3-dimensional cultures (3-fold) [97]	-
GSK1016790A	TRPV4 activator	Long-term systemic treatment with GSK1016790A significantly reduced the renal manifestations of PKD in a time-dependent manner. At the cellular level, GSK1016790A restored mechanosensitive Ca^+2^ signaling and improved both the function and the subcellular distribution of TRPV4 [21]	-
Niclosamide and Benzbromarone	Non-selective TMEM16A inhibitors. Block Cl^−^ currents and inhibit the expression of TMEM16A upon long-term treatment	The knockout of TMEM16A or the inhibition of TMEM16A in vivo using niclosamide and benzbromarone, significantly reduced cyst growth and abnormal cell proliferation [87]	-
Ani9	Selective TMEM16A inhibitors [98]	Ani9 significantly reduced cyst growth and abnormal cell proliferation in a PKD mouse model [87]	-
VX-809	Corrector of CFTR; VX-809 down-regulates cAMP levels, which reduced cell proliferation. It also affects adenylyl cyclase 3 activity, leading to a decrease in resting intracellular Ca^+2^ levels and the release of Ca^+2^ from the endoplasmic reticulum [99]	VX-809 at 30 mg/kg to mice or at 10 μm to cells did significantly inhibit cell proliferation when compared with control mice or cells [99]	-
Steviol	Inhibiting CFTR chloride channel activity and reducing CFTR expression [100]	Steviol inhibited the forskolin-stimulated apical chloride current in MDCK epithelium in a dose-dependent manner. Prolonged treatment with 100 µM steviol for 24 h significantly reduced this chloride current, partially by decreasing CFTR protein expression in MDCK cells [100]	-

PKD: polycystic kidney disease, MOA: mechanism of action, TRPV4: transient receptor potential vanilloid 4, CFTR: cystic fibrosis transmembrane conductance regulator, MDCK: Madin–Darby canine kidney.

## Data Availability

No new data were created or analyzed in this study.

## Abbreviations

**AC**	Adenylate cyclase
**ADPKD**	Autosomal dominant polycystic kidney disease
**ANG II**	Angiotensin II
**ARPKD**	Autosomal recessive polycystic kidney disease
**B-Raf**	B-rapidly accelerated fibrosarcoma
**Ca^+2^**	Calcium
**Camp**	Cyclic adenosine monophosphate
**CD**	Collecting duct
**CFTR**	Cystic fibrosis transmembrane conductance regulator
**ENAC**	Epithelial sodium channel
**ER**	Endoplasmic reticulum
**ERK**	Extracellular signal-regulated kinase
**ESRD**	End-stage renal disease
**FPC**	Fibrocystin
**IP3R**	Inositol 1,4,5-trisphosphate receptor
**MDCK**	Polarized Madin–Darby canine kidney
**MEK**	Mitogen-activated protein kinase
**NCCE**	Non-Capacitative Ca^+2^ Entry
**NHKS**	Normal human kidneys
**NO**	Nitric oxide
**PC1**	Polycystin-1
**PC2**	Polycystin-2
**PDEs**	Phosphodiesterases
**PKA**	Protein kinase A
**PKD**	Polycystic kidney disease
**RyR**	Ryanodine receptor
**TMEM16A**	Transmembrane member 16A anoctamin 1
**TRP**	Transient receptor potential
**TRPA**	Transient receptor potential ankyrin
**TRPC**	Transient receptor potential canonical
**TRPM**	Transient receptor potential melastin
**TRPML**	Transient receptor potential mucolipin
**TRPP**	Transient receptor potential polycystin
**TRPV**	Transient receptor potential vanilloid
**WGS**	Whole-genome sequencing
